# Tolerance through Education: How Tolerogenic Dendritic Cells Shape Immunity

**DOI:** 10.3389/fimmu.2017.01764

**Published:** 2017-12-11

**Authors:** Matthias P. Domogalla, Patricia V. Rostan, Verena K. Raker, Kerstin Steinbrink

**Affiliations:** ^1^Department of Dermatology, Division for Experimental and Translational Research, University Medical Center of the Johannes Gutenberg-University Mainz, Mainz, Germany; ^2^Research Center for Immunotherapy, University Medical Center of the Johannes Gutenberg-University Mainz, Mainz, Germany

**Keywords:** tolerogenic dendritic cells, regulatory T cells, immunotherapy, tolerance, nanoparticles

## Abstract

Dendritic cells (DCs) are central players in the initiation and control of responses, regulating the balance between tolerance and immunity. Tolerogenic DCs are essential in the maintenance of central and peripheral tolerance by induction of clonal T cell deletion and T cell anergy, inhibition of memory and effector T cell responses, and generation and activation of regulatory T cells. Therefore, tolerogenic DCs are promising candidates for specific cellular therapy of allergic and autoimmune diseases and for treatment of transplant rejection. Studies performed in rodents have demonstrated the efficacy and feasibility of tolerogenic DCs for tolerance induction in various inflammatory diseases. In the last years, numerous protocols for the generation of human monocyte-derived tolerogenic DCs have been established and some first phase I trials have been conducted in patients suffering from autoimmune disorders, demonstrating the safety and efficiency of this cell-based immunotherapy. This review gives an overview about methods and protocols for the generation of human tolerogenic DCs and their mechanisms of tolerance induction with the focus on interleukin-10-modulated DCs. In addition, we will discuss the prerequisites for optimal clinical grade tolerogenic DC subsets and results of clinical trials with tolerogenic DCs in autoimmune diseases.

## Introduction

The antigen-specific induction of immunological tolerance in the context of autoimmune and allergic diseases, which are driven by undesired immune responses against the body’s own or foreign antigens, has long been described as ultimate solution for the treatment of excessive immune activation. Nowadays, common treatment options are life-long, systemic immune suppression, which however may lead to serious side effects like chronic infections or malignant transformation. Therefore, various cell types have been investigated to establish permanent antigen-specific immune tolerance toward the causative triggers. Dendritic cells (DCs) as key players in controlling immune responses by either inducing immunity or establishing tolerance through interaction with multiple immune cells seem to be excellent candidates for the re-establishment of permanent antigen-specific tolerance. Since their discovery in 1973 by Ralph M. Steinman, several *in vitro* protocols have been established for the generation of potent, stable tolerogenic DCs whereof some have recently been used for the treatment of transplantation rejection, autoimmune and allergic disorders *in vivo*. In addition, to avoid *ex vivo* generation and modulation of DCs, DC-specific *in vivo* targeting, e.g., by antibodies or nanoparticle-based approaches, which can directly deliver immunomodulatory drugs to DCs, have emerged as a promising tool. In this review, we will outline the different protocols for generation of tolerogenic DCs, their mechanisms of tolerance induction, and summarize their use in preclinical and clinical settings.

## Role of DCs in Immunity and Tolerance

Recognition of DCs as professional antigen-presenting cells has come a long way. Antonio Lanzavecchia once stated that DCs seemed “too rare to be relevant” (1). With the Steinman lab pioneering DC immunology in the 1980s, the field started to expand rapidly and apart from their function in induction and maintenance of immunity, they also became relevant as promising candidates for immunotherapy with regards to tolerance induction.

Some refer to DCs as “nature’s adjuvants” highlighting their central role in the induction of immune responses. DCs populate almost all body surfaces in order to serve as sentinels detecting pathogens either by membrane-bound toll-like receptors (TLRs) or within the cytosol through nucleotide-binding oligomerization domain-like receptors (NLR) (2, 3). They do not kill the pathogen directly but use an even more sophisticated approach that induces long-lasting antigen-specific responses sufficiently bridging innate and adaptive immunity. By utilizing a proteolytic machinery (endolysosomal and proteosomal), they partially degrade antigens to peptides to subsequently display peptide/major histocompatibility (MHC) complexes on their surface (4). Although other cells such as macrophages and B cells are also able to present antigens *via* MHC, DCs are the only cell type to activate naïve T cells and to induce antigen-specific immune responses in all adaptive immune cells (4). They can for instance directly induce antibody production by presenting intact antigen to antigen-specific B cells without engaging T cells (5). DCs take a guiding role in immune responses as they interrogate, interpret, and transmit the nature of the antigenic stimulus, thereby shaping even T cell polarization *via* different intracellular signaling pathways (6).

Immature DCs (iDCs) are predominantly found in the peripheral tissues where they patrol and extensively take up large quantities of membrane-bound or soluble antigen by macropinocytosis and phagocytosis. However, at an immature state, DCs are inefficient in displaying MHC/peptide complexes on their surface as, e.g., their lysosomal activity is attenuated (3). The ability to channel MHC/peptide complexes to the surface increases upon engagement of pathogen recognition receptors such as TLRs or NLRs, which drive DC maturation (7). DCs change their capacity from antigen accumulation to T cell activation within only 1 day. Expression of chemokine receptors [C–C chemokine receptor (CCR) 1, CCR2, CCR5, CCR6, and C–X–C chemokine receptor (CXCR) 1] facilitates immature DC recruitment to the site of inflammation. Activation of DCs results in CCR6 downregulation and CCR7 and CXCR4 upregulation directing DCs toward the lymph node (8, 9).

Dendritic cell maturation, however, has a high degree of plasticity meaning that differentiated mature DCs (mDCs) can easily convert to tolerogenic DCs. This has been shown, e.g., by a group that stimulated activated DCs with pro-inflammatory interferon-γ (IFN-γ), which promoted the expression of indoleamine 2,3-dioxygenase (IDO) leading the respective DCs to acquire tolerogenic potential (10).

The original concept of tolerance induction by DCs is attributed to low amounts of surface MHC and co-stimulatory molecules such as cluster of differentiation (CD) 80 and CD86 found on iDCs. In contrast, the CD80/CD86^high^ expressing mature DC counterpart would rather activate effector T cells. However, in an uninfected individual, maintenance of self-tolerance is ensured by a continuous input of short-lived DCs that provide self-antigens in the lymphatic tissues. Notably, DCs isolated in the cold from germ-free mice show expression of co-stimulatory molecules and activate T cells to enter cell cycle (11). This indicates that the original view of tolerance induction is highly dependent on DCs’ mutual state of development and activation, as well as the surrounding microenvironment of cytokines and growth factors.

Dendritic cells in the thymus establish (central) self-tolerance by the display of self-antigens to developing T cells inducing T cell negative selection or Treg differentiation (12). Induction of peripheral T cell anergy and apoptosis, attenuation of effector and memory T cell responses, and the generation and activation of regulatory T cell (Treg) subpopulations has been attributed to a variety of tolerogenic DC subtypes (13–16).

Dendritic cell subtypes in humans can be characterized by anatomical localization and respective function. In steady state, blood DCs are immature precursors of tissue or lymphoid organ DCs. Epithelial tissues contain non-lymphoid or migratory DC subtypes (17). Lymphoid tissues harbor resident DC populations, which lack migratory capacities and play a role in retrieval of antigen and maintenance of antigen-specific immune responses (e.g., follicular DCs that recycle and “store” antigen for prolonged B cell activation in lymph node germinal centers) (18). Upon pathogen encounter and subsequent inflammatory state, the DC content of tissue and lymphoid organs is altered. Steady state DCs are diluted by CD14^+^ classical monocytes and precursors of inflammatory DCs. Blood DCs might also enter tissues *via* CD62 ligand (L) and CXCR3 expression, which allows extravasation (19).

**Figure 1:**
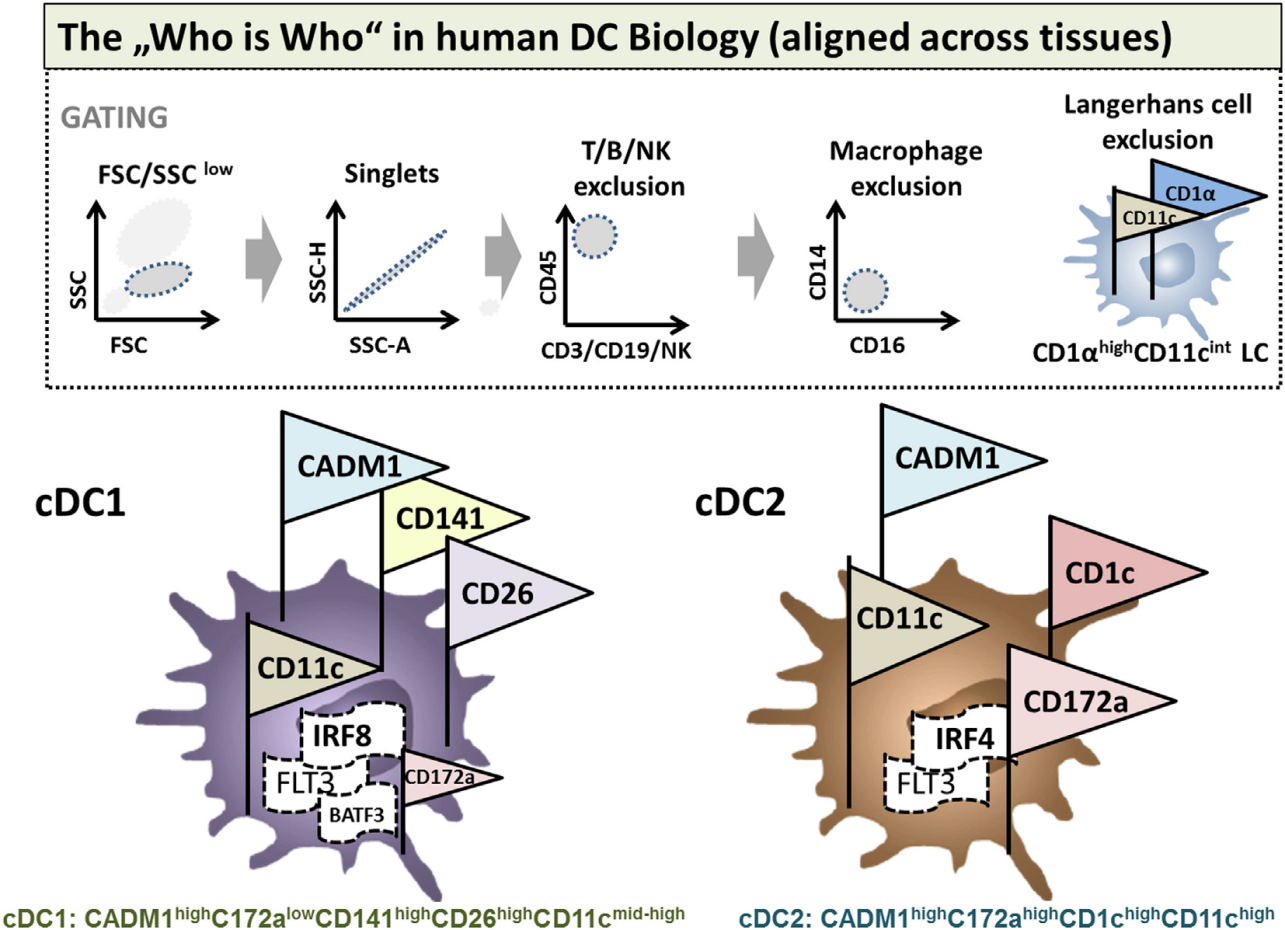
Flow cytometric phenotyping of dendritic cells (DCs) aligned across tissues. Surface marker expression of human and mouse DCs in a variety of tissues was defined previously by Guilliams et al. (22) for cDC1s and conventional type 2 DCs (cDC2s). To identify DC subpopulations, a multi-color FACS staining, FSC/SSC pre-gating, and linage (lymphocytes and NK cells) as well as macrophage exclusion has to be performed. If applicable for the desired tissue, afterward, CD45 immune cells are gated for CD1αhighCD11cint Langerhans cells (LCs). LCs excluded cells are then identified by the expression of either CADM1^high^C172a^low^CD141^high^CD26^high^CD11c^mid-high^ as cDC1s or CADM1^high^C172a^high^CD1c^high^CD11c^high^ cDC2s. In humans and mice, DC cell fate can be additionally identified on the level of transcription factors: DCs in general are dependent on flt-3. cDC1 development depends on BTAF3 and high levels of IRF8, whereas cDC2 evolution is dependent on IRF4 but independent of BATF3.

Site-specific appearance contaminating monocytes/macrophages and diverse inflammatory stimuli hinder a distinct phenotypical characterization of DC subsets. However, DCs were originally defined by their characteristic dendritic morphology and extraordinary capacity for antigen presentation and T cell priming (20, 21). These classical or conventional DCs (cDCs) are now classified into two main subsets, the CD11b^+^ and CD8^+^/CD103^+^ cDCs in mice and the corresponding blood dendritic cell antigen (BDCA)-1^+^ (CD1c^+^) and BDCA-3^+^ (CD141^+^) cDCs in humans. Beyond these subsets, however, a significant functional, genetic, and phenotypic diversity of DCs has been recently appreciated. There have been huge recent efforts by the scientific community to identify strategies in order to align DC phenotypes in a tissue and cross-species specific manner *via* flow cytometry. A set of lineage-imprinted markers recently published by Guilliams et al. is sufficient to differentiate between human plasmacytoid DCs [pDC: CD45^+^CD11c^low^ human leukocyte antigen-D related (HLADR)^high^ interferon response factor (IRF) 8^high^IRF4^mid^], conventional type 1 (cDC1s), and conventional type 2 DCs (cDC2s) (Figure 1). The authors provide a smart strategy to identify human cDC1s within (monocyte/macrophage excluded) CD14^−^CD16^−^ cells as cell adhesion molecule (CADM)1^high^CD172a^low^CD11c^mid/high^CD26^high^ cells and cDC2s as CADM1^low^CD172a^high^CD1c^high^CD11c^high^ cells validated by means of mass spectrometry and even on transcription factor level. This strategy is robust even under inflammatory conditions, in different tissues and allows identification of the same DC subset in macaques, humans, and mice (22).

A sophisticated identification strategy will allow for a more profound analysis of DC fates in mice and humans with regard to immunological functions of DCs in immunity and tolerance.

## Generation and Suppressive Mechanisms of Tolerogenic DCs

During classical immune responses, after encountering an antigen in combination with a danger signal, DCs upregulate the expression of co-stimulatory molecules, lymph node-homing receptors plus MHC molecules, and start the secretion of pro-inflammatory cytokines (21). Those processes enable DCs to migrate to the lymph nodes and initiate the activation of naïve T cells. Full T cell activation requires a three step signaling process. First, the binding of the T cell receptor (TCR) to its cognate antigen, which is presented on MHC molecules, second, the engagement of CD28 with co-stimulatory molecules like members of the B7 protein family CD80 and CD86, and third, interaction of DC-secreted cytokines with appropriate respective cytokine receptors (23).

**Figure 2:**
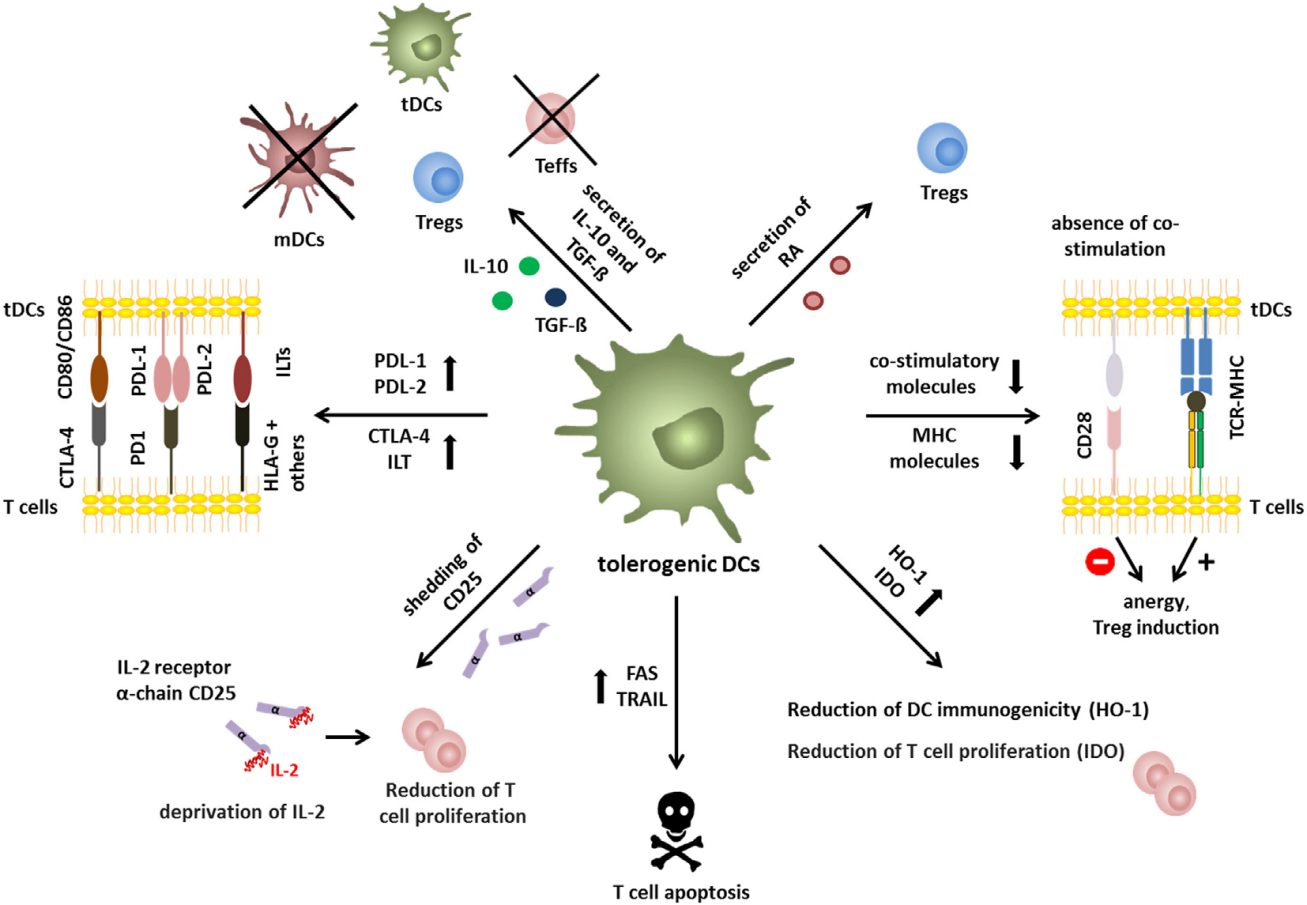
Immunosuppressive mechanisms of tolerogenic dendritic cells (DCs). Immunosuppressive mechanisms of tolerogenic DCs include secretion of immunomodulatory mediators, like interleukin (IL)-10 and TGF-β, or retinoic acid, resulting in induction of tolerogenic DCs, inhibition of effector T cell function, and Treg generation. In addition, absence or reduction of major histocompatibility and co-stimulatory molecules is involved in induction of anergic T cells with regulatory capacity. Furthermore, expression of immune-modulatory/-inhibitory molecules like PDL-1/-2, CTLA-4, and ILT-3/4 or expression of death receptors like TRAIL or FAS represent mechanisms to inhibit efficient T cell responses by tolerogenic DCs. In addition, deprivation of nutrition factors by the expression of indoleamine 2,3-dioxygenase (IDO) and heme oxygenase-1 (HO-1) results in reduced T cell proliferation and Treg induction, respectively. In a similar way, shedding of soluble CD25 leads to IL-2 deprivation and reduced T cell proliferation.

In contrast, tolerogenic DCs exploit several immunosuppressive mechanisms to induce tolerance (Figure 2). Tolerogenic DCs often display an immature or semi-mature phenotype that is characterized by low expression of co-stimulatory and MHC molecules and altered cytokine production. Presentation of low levels of antigen without co-stimulation leads to T cell anergy (24) and promotion of regulatory T cell differentiation *in vitro* and *in vivo* (25–27). TCR signaling in combination with co-stimulation results in activation of the transcription factors nuclear factor of activated T cells (NFAT), activator protein (AP)-1, and nuclear factor “kappa-light-chain-enhancer” of activated B-cells (NF-κB) that subsequently induce a transcriptional program resulting in T cell activation (28). It is not yet exactly clear how absence of co-stimulation results in a transcription profile that favors Treg induction but impaired CD28-induced activation of the rat sarcoma/mitogen activated protein kinase (Ras/MAPK) pathway results in deficient AP-1 activation. In the absence of AP-1, NFAT proteins, possibly in combination with other transcription factors or by forming dimers, may subsequently initiate a transcriptional program that cumulates in T cell anergy and Treg induction (29). However, recent studies demonstrated that phenotypically mDCs are also capable of inducing Tregs, indicating that the phenotype does not necessarily determine the immunogenic or regulatory function of DCs (13, 30, 31). Furthermore, secretion of anti-inflammatory cytokines like interleukin (IL)-10 and transforming growth factor-β (TGF-β) and reduced expression of pro-inflammatory cytokines by DCs critically contribute to tolerance induction. Production of IL-10 by tolerogenic DCs is indispensable for regulatory function in multiple settings (32–34) and DC-released TGF-β is important for tolerance induction as DC-specific ablation of the TGF-β activating integrin αvβ8 (Itgb8) results in autoimmunity and colitis as demonstrated in transgenic CD11c-Cre/Itgb8^fl/fl^ mice (35). Moreover, TGF-β secretion by tolerogenic DCs is important for the regulation of T_H_17 responses in neuro-inflammation as shown in CD11c^DNR^ mice, which is a dominant-negative form of TGF-β receptor II resulting in diminished TGF-β signaling (36). Furthermore, IL-10 and TGF-β, which are secreted by tolerogenic DCs in the tumor microenvironment, facilitate and reinforce tumor escape (37). In addition, several immunosuppressive features of tolerogenic DCs rely on induction of apoptosis in responding T cells including Fas cell surface death receptor (FasL/Fas) interactions (38) and tumor necrosis factor-related apoptosis-inducing ligand (TRAIL)/TRAILR engagement (39). Tolerogenic DCs may also express various inhibitory receptors like for example programmed cell death ligand (PDL)-1, PDL-2 (40, 41), inhibitory Ig-like transcripts (ILT) (42, 43), and cytotoxic T-lymphocyte-associated protein 4 (CTLA-4) (44), which act on T cells by dampening TCR signaling and competing with CD28, respectively. Tolerogenic DCs also alter T cell responses by modulation of metabolic parameters for example by the release of IDO and the induction of heme oxygenase-1 (HO-1) to control levels of tryptophan and carbon monoxide. IDO facilitates the degradation of tryptophan to N-formylkynurenin leading to reduced T cell proliferation (45, 46), whereas HO-1 inhibits hemoglobin, resulting in production of carbon monoxide, which leads to reduced DC immunogenicity (47, 48). In addition, tolerogenic DCs are also capable of producing retinoic acid (RA) (49), inducing Treg differentiation (50). Shedding of CD25 by DCs and subsequent deprivation of IL-2 was recently proposed as additional immunosuppressive mechanism for the suppression of effector T cell proliferation (13).

Several human tolerogenic DC subsets have been characterized *in vitro* based on their tolerogenic capacities. iDCs display minimal expression of co-stimulatory molecules and no secretion of inflammatory cytokines, demonstrating the aforementioned optimal requirements for tolerance induction, which has also been demonstrated *in vitro* (51). However, iDCs are unstable and may differentiate into immunogenic DCs under inflammatory conditions (13, 52).

**Figure 3:**
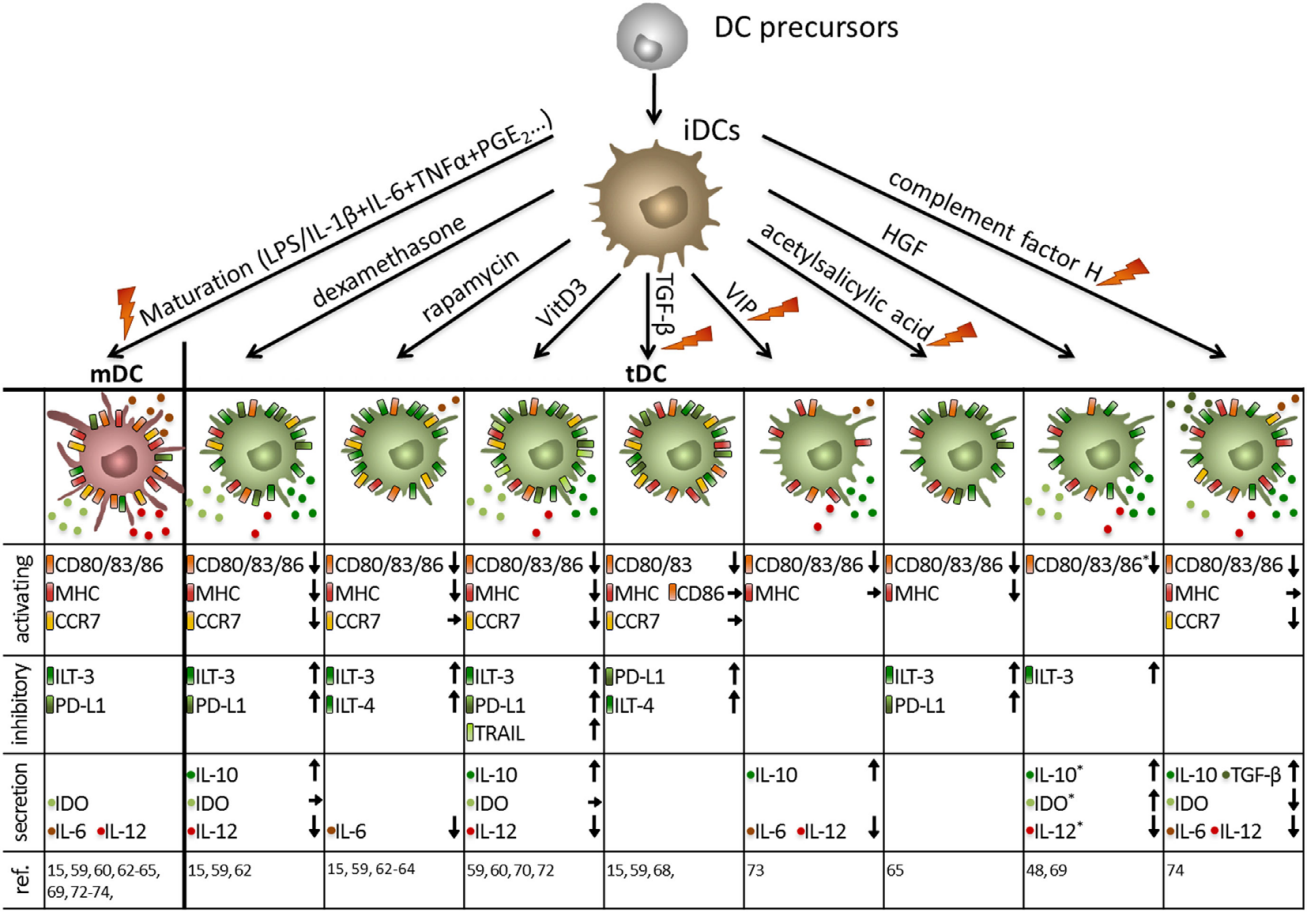
Human tolerogenic dendritic cells (DCs) are induced by various immunosuppressive drugs and mediators. Immuno-activating and -inhibitory surface molecules as well as secreted signaling molecules are demonstrated. Arrows indicate up/downregulation or unchanged expression or secretion by human tolerogenic DCs compared to either mature DCs (activating surface molecules + secretion) or immature DCs (iDCs) (inhibitory surface molecules), respectively. As an exception, expression of activation molecules, and secretion of immune mediators marked with * are compared to iDCs. Note: in some protocols, tolerance-inducing agent is added at the beginning and during the entire culture of tolerogenic DCs, whereas others are added at the end of the culture for 1–3 days either with or without a maturation stimulus. Protocols that involve a maturation stimulus are marked with an orange flash. *Compared to iDCs.

Therefore, many protocols have been established to generate stable human DCs with tolerogenic capacities *in vitro* (Figure 3). The opportunity to genetically modify human DCs has been exploited to directly induce tolerogenic properties by the recombinant expression of FasL (53), PD-L1 plus TRAIL (54), or IDO (55) all of which lead to the induction of T cell apoptosis and suppression of effector T cell function, respectively. Additionally, DCs can genetically be engineered to secrete enhanced levels of IL-10 (56) or TGF-β (57) resulting in broad immunosuppression.

Furthermore, human tolerogenic DCs are induced by various immunosuppressive drugs (Figure 3) that are often systemically used to control excessive immune responses like corticosteroids, rapamycin, cyclosporine, or by acetylsalicylic acid (58). For instance, the corticosteroid dexamethasone is capable of inducing tolerogenic DCs that exhibit low expression of co-stimulatory molecules combined with highly expressed inhibitory receptors ILT-2 and ILT-3 and secrete large amounts of IL-10 and IDO resulting in the induction of T cells with regulatory capacities (15, 59–61). In a similar way, the immunosuppressive drug rapamycin, which inhibits mechanistic target of rapamycin persuades human DCs to express a stable tolerogenic phenotype with reduced expression of MHC and co-stimulatory molecules in combination with a high ILT-3 and ILT-4 expression, leading to Treg generation *in vitro* and *in vivo* (15, 59, 62–64). Furthermore, in the presence of acetylsalicylic acid, DCs downregulate the expression of co-stimulatory molecules, whereas inhibitory molecules like ILT-3 and PD-L1 are upregulated resulting in Treg induction (65).

**Figure 4:**
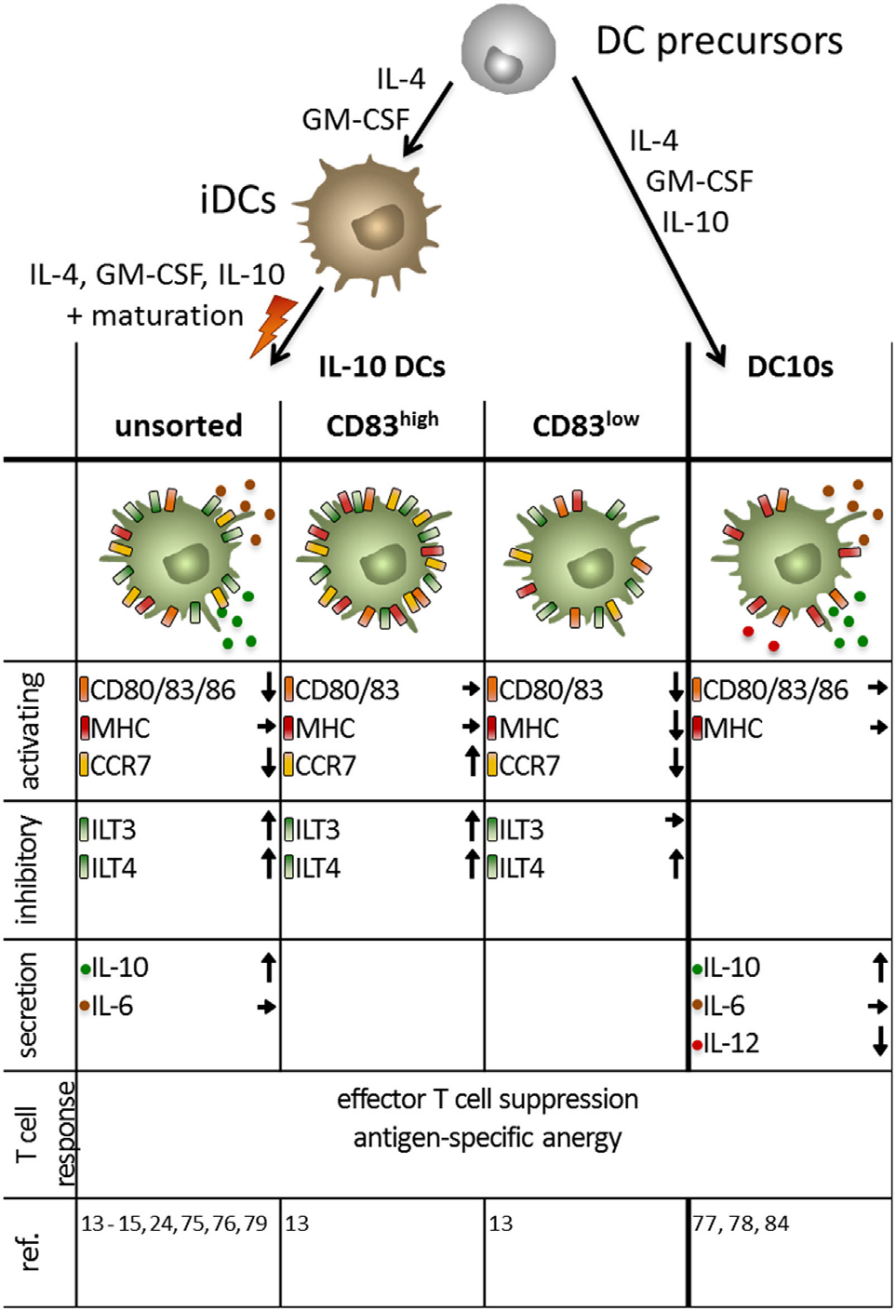
Phenotype of monocyte-derived interleukin (IL)-10 dendritic cells (DCs) obtained by different protocols. Immuno-activating and -inhibitory surface molecules as well as secreted signaling molecules and the T cell response are depicted. Arrows indicate up/downregulated or unchanged expression or secretion by human IL-10-modulated tolerogenic DCs compared to mature DC. IL-10 DCs are generated by addition of the immunosuppressive cytokine during the maturation step at the end of the culture, whereas DC10 are obtained by incubation with IL-10 during the entire culture period.

In addition, incubation with the immunosuppressive cytokines TGF-β and IL-10 alone or in combination facilitated the generation of a tolerogenic DC phenotype (Figures 2–4) (48, 66). For instance, TGF-β dampens the antigen-presenting capabilities of DCs by downregulation of MHC and co-stimulatory molecules and upregulation of PDL-1 resulting in T cell anergy (15, 36, 59, 67, 68). The comprehensive tolerogenic properties of IL-10 induced tolerogenic DCs will be discussed in detail in the next chapter. However, other bioderivates are also capable of inducing DCs with tolerogenic function (Figure 3). In the presence of hepatocyte growth factor, DCs express various tolerance-inducing molecules like IDO, IL-10, TGF-β and TRAIL, which may induce T cell with regulatory functions (48, 69). Furthermore, treatment of human DCs with vitamin D3 (VitD3) triggers a tolerance-inducing phenotype that is characterized by enhanced IL-10 secretion, augmented IDO production and the expression of PDL-1 and Trail. Resulting DC populations are either capable of inducing antigen-specific T cell apoptosis or expansion of Tregs (59, 60, 70–72). The immunoregulatory neuropeptide vasoactive intestinal peptide prevents full maturation of DCs and induces high IL-10 secretion (73). Furthermore, a recent study by Olivar et al. demonstrated tolerogenic capacities of human DCs that are generated in the presence of the complement factor H resulting in reduced expression of co-stimulatory molecules on DCs, enhanced IL-10 and TGF-β gene expression and induction of forkhead box P3 (FOXP3)^+^ Tregs (74).

Since a comparative study by Boks et al. in 2012 identified human IL-10-modulated tolerogenic DCs (IL-10 DCs) as the most potent candidates for antigen-specific induction of tolerance *in vivo* (15), their generation and suppressive mechanisms will be highlighted in the next chapter.

## Mechanism of Tolerance Induction by Human IL-10-Modulated Tolerogenic DCs

Interleukin-10 DCs have been playing a pivotal and central role in the research field of tolerogenic DCs for over two decades. However, a variety of *in vitro* protocols exist for the generation of IL-10 DCs leading to a huge amount of data that are challenging to compare (Figure 4).

Interleukin-10-modulated DCs are usually generated from monocytes when cultured in the presence of IL-4 and GM-CSF to induce iDCs. The two most prominent protocols add IL-10 either during the whole culture (referred to as DC10s in the following) or at a later time point together with a maturation stimulus (referred to as IL-10 DCs) (13–15, 24, 75–79).

Gregori and colleagues generated DC10s using the first mentioned protocol and characterized them as CD14^+^CD16^+^CD11c^+^CD11b^+^CD40^+^HLA-DR^+^CD80^+^CD83^+^CD86^+^CD163^+^ and CD1a^−^CD1c^−^CD68^−^CD115^−^MDC8 DCs. In contrast, mDCs are CD14^−^ and CD16^−^, but express comparable amounts of CD80, CD83, CD86, and HLA-DR (77). It can be argued that the high expression of CD14 and CD16 indicate a macrophage-like phenotype (80). However, the lack of expression of the monocyte marker CD115 and the macrophage marker CD68 in combination with the constitutive expression of CD83, a DC-associated molecule, identified DC10s as immune cells of the DC lineage. Moreover, they show a DC-like morphology and are capable of driving naïve T cells to develop into antigen-specific Tr1 cells (77). DC10s also express the co-inhibitory molecules ILT-2, ILT-3, ILT-4, and HLA-G, and their capability to induce anergic Tr1 cells is ILT-4 dependent (77). Amodio et al. stated that the expression of HLA-G on DC10s is donor dependent and correlates with the expression of ILT-4 and with the frequency of Tr1 induction (78). These Tr1 cells are CD49b^+^ and LAG3^+^ and secrete high levels of IL-10, low IL-2, no IL-4, no IL-17, and variable amounts of IFN-γ (81–83). After LPS and IFNγ stimulation, mDCs and DC10s secrete comparable amounts of pro-inflammatory IL-6, but neither secrete the T_H_1-inducing cytokine IL-12, yet, DC10s produce slightly higher amounts of TNFα and considerably more IL-10 (77). The major function of tolerogenic DCs, the induction of Tregs, was found to be dependent on IL-10 secretion (77), similar to the upregulation of HLA-G on CD4^+^ T cells through stimulation with DC10s (78).

Gregori et al. have also identified the *in vivo* counterpart of the *in vitro* generated DC10s as naturally occurring DC10s in humans. They were sorted from peripheral blood from healthy volunteers as CD14^−^CD11c^+^CD83^+^ and analyzed for their poststimulation cytokine profile. In accordance with the findings from monocyte-derived DCs, their IL-12 secretion was negligible, but they produce relevant amounts of IL-6 and TNFα. Most importantly, their IL-10 levels were significantly increased compared to iDCs and mDCs (77).

A slightly different phenotype can be observed, when IL-10 DCs are generated using the latter previously mentioned protocol in which IL-10 is added for the last 2 days of the culture during the maturation step (Figure 4). Here, presence of IL-10 prevents full DC maturation, indicated by intermediate expression of the co-stimulatory molecules CD80 and CD86, as well as the DC maturation marker CD83 (13, 75). The tolerogenic phenotype is further established through the increased expression of the co-inhibitory molecules ILT-3 and ILT-4 (13, 15). This IL-10 triggered surface marker modulation is dependent on glucocorticoid-induced leucin zipper, a transmembrane molecule, which blocks NF-κB, MAPK, and AP-1 (84). The results for the expression of HLA-DR and CD14 on IL-10 DCs are contradictory. Our studies revealed that the whole IL-10 DC population shows an intermediate HLA-DR expression and that a subpopulation of IL-10 DCs are CD14^+^ (24), whereas other groups found that HLA-DR expression is comparable to mDCs and IL-10 DCs are exclusively CD14^−^ (85). In comparison with mDCs, IL-10 DCs stimulate a reduced T cell activation and are capable of inducing an antigen-specific anergy in CD4^+^ or CD8^+^ naïve T cells (24, 75, 76). The induction of anergy is associated with the increased expression of the MAPK p38 and its effector molecules MAPK-activated protein kinases 2 and 3 (86) as they upregulate the expression of the cyclin-dependent kinase inhibitor 1B (p27^Kip1^), leading to a cell cycle arrest in the G1 phase (79). The induced Tregs in turn have the ability to efficiently suppress syngeneic effector CD4^+^ and cytotoxic CD8^+^ T cells in a cell-to-cell contact-dependent and antigen-specific manner (15, 24, 79).

IL-10 DCs were identified as the most suitable candidate for DC-mediated tolerance-vaccination therapies as was shown by a comprehensive study by Boks et al. They compared five protocols for *ex vivo* induction of human tolerogenic DCs (through VitD3, dexamethasone, TGF-β, rapamycin, and IL-10) with regard to prerequisites for clinical applications in humans such as potent migratory capacity, sufficient Treg induction, and the stability of the tolerogenic phenotype under inflammatory conditions to guarantee the safety of the therapy (13, 15). The protocol using IL-10 for tolerogenic DC generation was shown to be superior as compared to the other tested protocols, with respect to the stability of the tolerogenic phenotype and the suppressive capacity of the induced Tregs (15). Boks et al. also revealed that co-maturation was indispensable for the stability of the phenotype and for the migratory capability in all protocols tested (15).

However, in their study, IL-10 DCs displayed a limited migratory capability due to a reduced CCR7 expression (15). This was confirmed by another comparative study by Adnan et al., which compared tolerogenic DC protocols in a similar way. However, they also showed that IL-10 DCs induce higher numbers of IL-10^+^CD4^+^ Tregs than tolerogenic DCs generated with other protocols [involving protein kinase C inhibitor (PKCI), VitD3, dexamethasone, TGF-β, rapamycin, and peroxisome proliferator-activated receptor γ + all-trans RA] and that the Tregs induced by both IL-10 and PKCI-treated tolerogenic DCs exhibited a higher suppressive capacity compared to Tregs induced by other tolerogenic DC protocols (13, 15, 59, 84, 85). In accordance with that, among all protocols tested by Boks et al., only IL-10 DC-induced Tregs exhibited a significantly enhanced suppressive function, compared to other tolerogenic DCs. Therefore, Boks et al. concluded that IL-10 DCs are the most suitable candidates for tolerogenic DC-based therapies for allergic and autoimmune diseases and transplantation rejections (15).

Recent investigations of our own laboratory refined this thesis by identifying two subpopulations of the human tolerogenic IL-10 DCs, distinguishable by the expression of CCR7 and CD83 (CD83^high^CCR7^+^ and CD83^low^CCR7^−^ IL-10 DCs) (13). Both IL-10 DC subsets were capable of inducing Tregs, but the CD83^high^ IL-10 DC-induced Tregs exhibited a significantly enhanced suppressive capacity. It is evident from the proliferation, cytokine production, and surface makers that Tregs induced by CD83^high^ IL-10 DCs exhibit a more activated phenotype compared to Tregs induced by CD83^low^ IL-10 DCs. In addition, the tolerogenic phenotype of the CD83^high^ IL-10 DC population was found to be extremely stable in the presence of IL-1β, IL-6, and TNFα, mimicking an inflammatory environment (13). In contrast to mDCs, IL-10 DCs and predominantly the CD83^high^ subpopulation express increased amounts of membrane-associated and soluble CD25, the latter of which was found to play a role in the suppression of T cell proliferation (13). CD25 is known to exert seemingly contradicting functions: the membrane-bound molecule may be involved in the stimulation of T cells, whereas the soluble form attenuates T cell proliferation by trapping IL-2 (87, 88).

However, most importantly, dependent on their high expression of CCR7, the CD83^high^ IL-10 DCs displayed a pronounced migratory capability that is superior to that of CD83^low^ or unsorted IL-10 DCs (13, 81, 89). Therefore, in conclusion, the tolerogenic characteristics of the most promising population of tolerogenic DCs, IL-10 DCs can be further improved by sorting for CD83^high^ IL-10 DCs.

## Nanoparticle-Based *In Vivo* Induction of Tolerogenic DCs

The above discussed protocols greatly expanded the knowledge of tolerogenic DC biology and enabled scientists to generate tolerogenic DCs that are stable under inflammatory conditions and may be used for antigen-specific clinical application. However, for this purpose, DC precursors need to be isolated from the patient’s blood, modulated *ex vivo* and re-injected into the patient, which is a time-consuming and expensive process. In addition, recent data suggest that monocyte-derived DCs, which are used in such immunotherapeutic approaches may rather be allocated to the family of monocytes, which have less T cell stimulatory capacities than DCs *in vivo* (90, 91). Therefore, nanoparticle-based drug delivery systems that enable directed cell-type specific targeting *in vivo* in combination with delivery of multiple drugs in one formulation have emerged as another promising approach in DC-based immunotherapy.

For cell type-specific targeting, nanoparticles can be chemically conjugated to antibodies, peptides, carbohydrates, or cytokines that address receptors that are preferentially expressed on DCs (92–95). For instance, targeting of human DCs *in vivo* with subsequent antigen presentation and robust humoral and cellular responses can be achieved by antibodies against the c-type lectin receptor DEC205 as shown in a recent phase 1 clinical trial (96). In addition, other possible receptors that have been used to specifically target DCs include DC-SIGN, the mannose receptor, Fc receptors, CD40, or CD11c (93, 97, 98). Even though most approaches focus on induction of immunity for example in the context of tumor immunotherapy, cell-type-specific nanoparticle delivery is also a promising strategy to prevent excessive immune responses and induce DCs with tolerogenic capacity. For instance, polymeric synthetic nanoparticles that target DCs have been used to induce OVA-specific tolerance by delivery of rapamycin (99). In a similar approach, Zhang et al. were able to prevent antibody formation against substituted factor VIII (100). Intriguingly, Clemente-Casares et al. generated nanoparticles that target disease relevant peptides toward MHC II molecules, which subsequently trigger the expansion of antigen-specific Tr1 cells and regulatory B cells in different autoimmune disease models such as type 1-diabetes, inflammatory bowel disease, rheumatoid arthritis, and multiple sclerosis resulting in alleviation of disease symptoms (101).

## Use of Tolerogenic DCs in Clinical Applications

**Figure 5:**
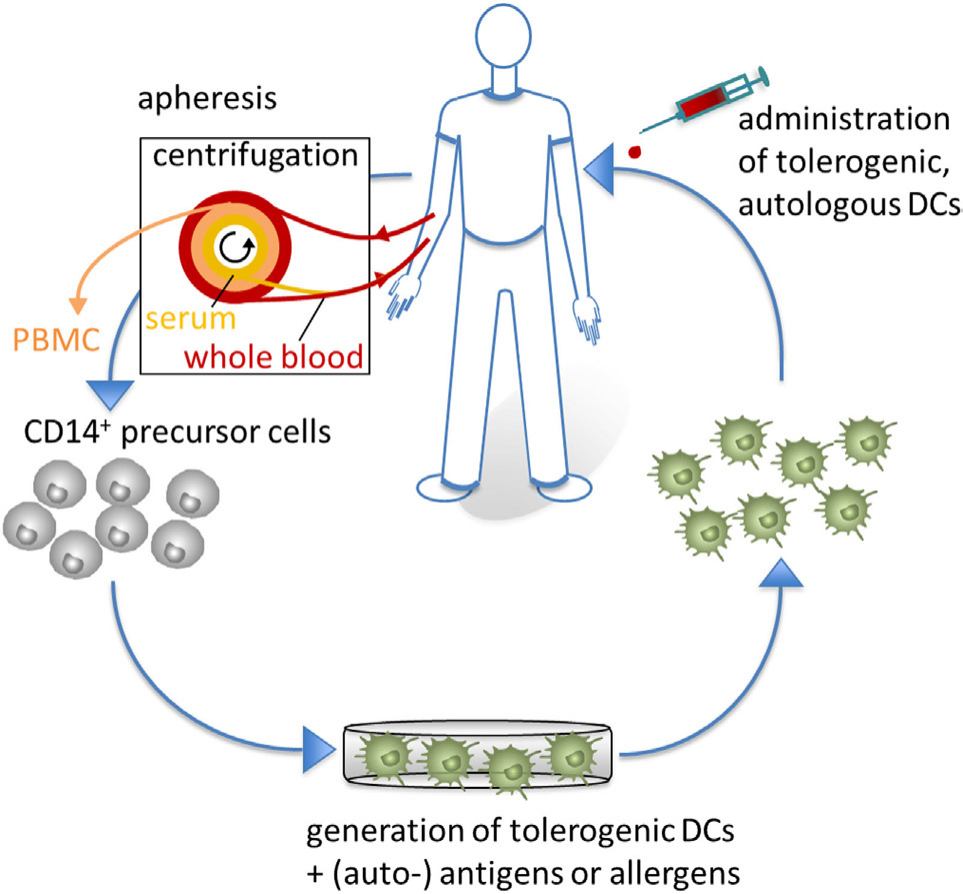
Tolerogenic dendritic cells (DCs) in clinical application. For clinical applications, CD14^+^ monocytes are isolated from apheresis products to generate tolerogenic DCs, which are loaded with (auto-) antigens or allergens. Subsequently, antigen-specific tolerogenic DCs are reinjected into the patients to affect the inflammatory immune response of autoimmune or allergic diseases.

Over the last decades, numerous trails with DC-based immunotherapies have been conducted using activated, mDCs to stimulate antitumor immune responses, and some have shown objective clinical benefits in patients with different types of cancer, including prostate cancer or malignant melanoma (102–104). Currently, several immunotherapeutic approaches are being studied using tolerogenic human DCs for treatment of inflammatory, autoimmune, and allergic diseases as well as transplant rejections (58, 105) (Figure 5).

In contrast to standard immunosuppressive therapies, which often do not specifically target the cause of disease and are accompanied by severe side effects, *ex vivo* generated tolerogenic DCs may be an attractive therapeutic approach to induce, enhance, or restore (antigen-specific) tolerance. After loading with exogenous or endogenous antigens, one major advantage of tolerogenic DC vaccination is their capability to act in an antigen-specific manner.

Evidence from several rodent models clearly showed the efficacy of tolerogenic DCs in the fields of inflammatory, autoimmune, and allergic disorders and transplantation medicine (58, 105). To translate the results into the human system, several *ex vivo* studies have been performed as proof of principle experiments demonstrating that human tolerogenic DCs efficiently inhibit disease-related immune responses, e.g., by induction of Tregs or T cell anergy and apoptosis. With regard to allergic diseases, *ex vivo* models have shown that human tolerogenic IL-10 DCs from atopic donors suppressed T_H_2 immune responses by induction of FOXP3^+^ Tregs and dexamethasone-induced tolerogenic DCs activated IL-10 producing Tregs, specific for the latex Hev b 5 antigen, in rubber latex allergic patients (106, 107). Since tolerogenic DCs are also a promising tool to restore tolerance to specific tissue-derived autoantigens, several *ex vivo* studies have been conducted with tolerogenic DCs obtained from patients suffering from autoimmune disorders (58). Tolerogenic VitD3-treated DCs derived from precursor cells of multiple sclerosis patients and loaded with myelin peptides induced a stable and antigen-specific hyporesponsiveness of autologous T cells (58, 108), which was shown to be TGF-β-dependent (109). Studies in type 1 diabetes patients revealed that tolerogenic DCs, generated either in the presence of vitamin D or of IL-10/TGF-β, and loaded with the pancreatic islet antigen glutamic acid decarboxylase 65 rendered antigen-specific T cells hyporesponsive toward a second challenge with fully competent, antigen-loaded DCs (66, 110). Furthermore, monocyte-derived DCs were obtained from systemic lupus erythematosus (SLE) patients, treated with dexamethasone/rosiglitazone and loaded with self-antigens. Those tolerogenic DCs can modulate CD4^+^ T cell activation and are a suitable tool for antigen-specific immunotherapy in SLE (111).

Although safety and feasibility of DC-based studies in general have already been shown, there are still a lot of open questions regarding the DCs manufacturing protocols, the route of application, the numbers of DCs, and the frequency and time points of injections. In addition, the characteristics of tolerogenic DCs, including the phenotype, migratory capacity, stability under inflammatory conditions, and the mode of action (induction/activation of regulatory T and B cells, T cell anergy and apoptosis induction, interaction with other immune cells) have to be investigated and different protocols have to be compared with regard to these properties. Aiming to joint efforts in translating tolerogenic DCs into the clinic by harmonizing protocols and defining functional quality parameters, international co-operations in science, and technology network have been initiated (112, 113).

One major concern in the context of tolerogenic DC-based immunotherapies is the stability of the tolerogenic phenotype under inflammatory conditions as DCs express several pattern-recognition receptors and receptors for growth factors and cytokines, which can be stimulated in an inflammatory environment. Therefore, clinical grade tolerogenic DCs must be intensively tested for a robust, stable phenotype to exclude a loss of the regulatory function and a switch to an immunostimulatory phenotype of the differentiated DCs, leading to an (antigen-specific) immune activation rather than to the intended immunosuppressive reaction. Comparative studies revealed that most of the tolerogenic *ex vivo* generated DC populations (by use of, e.g., IL-10, TGF-β, VitD3, rapamycin, dexamethasone, PKCI as described above), exhibit the aforementioned stable phenotype (15, 59). However, both reports demonstrated that tolerogenic IL-10 DCs showed the most powerful tolerogenic properties in terms of Treg induction with strong suppressive capacities. Another important feature is the CCR7-directed migratory capacity of tolerogenic DCs toward secondary lymphatic organs, resulting in the induction and generation of T cell-mediated immunosuppression. A recent study (as mentioned above) revealed that IL-10 DCs are consisting of two different populations, CD83^high^CCR7^+^ IL-10 DC and CD83^low^CCR7^−^ IL-10 DC subpopulations, both exhibiting tolerogenic properties, resulting in Treg induction (13). However, sorting of IL-10 DCs into these two subsets ascertained a significantly improved migratory capacity of the CD83^high^CCR7^+^ IL-10 DC subpopulation compared to CD83^low^CCR7^−^ IL-10 DCs, and to the non-separated IL-10 DC population as well. The stable phenotype, efficient CCR7-directed migration, and, in particular, pronounced tolerogenic capacity to induce Tregs with high suppressive activity of IL-10 DCs is a prerequisite for clinical grade DCs considered for vaccinations studies in humans.

Regarding the route of DC administration, different applications have been used in humans. Tolerogenic DCs have been injected intraperitoneally in patients suffering from Crohn’s disease (114), intradermally in diabetes, and rheumatoid arthritis patients (115, 116), subcutaneously in rheumatoid arthritis patients (117) and *via* arthroscopic injections in joints of patients with rheumatoid or inflammatory arthritis (118). In all studies, the route of administration has been well tolerated without any signs of toxicity. Likewise reports of intravenous injections of tolerogenic DCs into nonhuman primates revealed their safety (119).

The first attempt to apply tolerogenic DCs to humans was undertaken by Ralf Steinman’s group in 2001 (120, 121). They showed that subcutaneous applications of human immature tolerogenic DCs (2 × 10^6^), generated in the presence of IL-4 and GM-CSF and pulsed with antigens, into healthy subjects was well tolerated and suppressed antigen-specific CD8^+^ T cell responses up to 6 months. Thus, they pioneered to demonstrate the tolerogenic potential of DCs in humans *in vivo*.

Several protocols for tolerogenic DCs have been tested in phase I trials with highly encouraging results from a safety point of view and in terms of adverse effects such as allergic reactions, exacerbations of autoimmunity, and pro-inflammatory immunity (114–118) (Table 1).

**Table 1:** Use of tolerogenic dendritic cells in clinical applicatsions.

Study	Indication	Patients	Protocol for tolDC	Antigen	Treatment regime	Route of application	Summary	Reference
Phase 1 randomized controlled	Type l diabetes	10 (5/5) insulin-requiring type 1 diabetic patients	Un-manipulated Antisense ODN targeting CD40, CD80, CD86	No antigen	1 × 10^6^ DC four times, every 2 weeks	Intradermal	No adverse effects, increase of B220^+^ CD11c^−^B cells, no change in other immune cell populations/biomarkers	(115)

Phase I	Rheumatoid arthritis	12	CeaVax-retinoic acid (RA)	Protein arginine deiminase 4, RA33, citrullinated fillagrin, vimetin antigens	0.5 × 10^7^ or 1.5 × 10^7^, five times at 2- to 4-week intervals	Subcutaneous	Grade 1 or 2 adverse effects, significant decrease in antigen-specific autoantibodies (55.6%) and IFN-γ-secreting t cells (91.7%), EULAR response of 83.3% of patients injected with high dose	(117)

Phase I randomized controlled	Rheumatoid arthritis	34 (18 treated/16 left untreated) HLA-DR risk genotype-positive RA patients	Bay 11-7082 (NF-κB inhibitor)	Citrullinated peptides: collagen type II fibrinogen α fibrinogen β vimentin	0.5–1 × 10^6^ or 2.0–4.5 × 10^6^ one injection	Intradermal	Grade 1 adverse effects, increased ratio of regulatory to effector T cells, reduction in serum IL-15, IL-29, CX3CL1, and CXCL11; reduced antigen-specific T cell responses (*p* < 0.05)	(116)

Phase I escalating	Crohn’s disease	12 (2 per cohort)	Dexamethasone and vitamin A	No antigen	2 × 10^6^, 5 × 10^6^ or 10 × 10^6^ once or three times (biweekly) in escalating doses	Intraperitoneal	No adverse effects, decrease in Crohn’s Disease Activity Index (CDAI) (*p* = 0.3) and Crohn’s Disease Endoscopic Index of Severity (*p* = 0.4), lesions improved markedly in three patients (33%)	(114)

Phase I escalating randomized controlled	Rheumatoid arthritis	12 [3 per treatment group (=9); saline control (=3)]	Dexamethasone and vitamin D3	Autologous synovial fluid	1 × 10^6^, 3 × 10^6^, and 10 × 10^6^	Intraarticular	Stabilized symptoms in two patients receiving 10 × 10^6^ to lDC, but no decrease in disease activity score 28 detectable; no immunomodulatory effects	(118)

The first clinical trial with tolerogenic DCs was carried out in 10 patients suffering from diabetes type 1 in 2011. They were injected intradermally four times at 2-week intervals with 1 × 10^7^ autologous DCs which have been either un-manipulated (controls) or have been treated with antisense oligonucleotides targeting CD40, CD80, and CD86 to silence these surface molecules. DC treatment was well tolerated without any adverse effects and did not induce autoantibody production (115). Analysis of the immune response revealed no alterations with exception of increased IL-4/IL-10 levels and elevated frequencies of a regulatory B220^+^CD11c^+^ B cell population. Importantly, the patients did not lose their ability to mount T cell responses, e.g., to pathogens, demonstrating the absence of a general immune suppression.

Another clinical trial was conducted to analyze the impact of tolerogenic DCs in nine patients suffering from Crohn’s disease (114). Here, under an escalating protocol tolerogenic, DCs (treated with dexamethasone and VitD3) were intraperitonally injected in once or biweekly intervals, respectively. The DC vaccination was well tolerated and did not induce adverse effects from week 1 to 12 and in a follow-up up to 12 months.

In the field of rheumatoid arthritis, three trials have been published to date. In one study, 12 patients were subcutaneously injected with a low (0.5 × 10^7^) or high dose (1.5 × 10^7^) of autologous DCs for five times at 2- to 4-week intervals (117) (Table 1). The tolerogenic DCs were pulsed with protein arginine deiminase 4, heterogeneous nuclear ribonucleoprotein A2/B1 (RA33), citrullinated filaggrin, and vimentin antigens (=CreaVax-RA). The authors observed only a few patients with grade 1 or 2 adverse effects, but a combination of a significant decrease in autoantibody levels and a good-to-moderate EULAR response at 14 days after initiation of the trial, which was more pronounced in the DC high-dose group. Bell et al. reported the results of another dose escalation trial (AUTODEKRA trial) with rheumatoid arthritis patients who were intra-articularly treated with tolerogenic DCs (1 × 10^6^, 3 × 10^6^, or 10 × 10^6^), generated in the presence of dexamethasone and VitD3 and loaded with autologous synovial fluid as source of autoantigens (118). No target knee flares and other severe side effects were observed. The authors did not find any trends in disease activity scores (DASs) or in consistent alteration of immune parameters in the peripheral blood; however, patients with the highest dose exhibited an improvement of the clinical symptoms.

In the study of Benham et al., tolerogenic DCs were generated in the presence of an NF-κB inhibitor, resulting in CD40 deficient but highly CD86 expressing tolerogenic DCs, which were administered to rheumatoid arthritis patients (116). For an antigen-specific immune response, DCs were pulsed with four different citrullinated peptide antigens (Rheumavax). 18 patients were injected intradermally with a single dose of the tolerogenic DCs (either 1 × 10^6^ or 5 × 10^6^). Evaluation of the patients after 1, 3, and 6 months revealed that the vaccination was well tolerated and no side effects in form of (auto-) inflammatory reactions have been observed. The authors found a reduction in effector T cells and several inflammatory mediators and an increased regulatory to effector T cell ratio in the patients. In addition, the DAS was decreased within 1 month in vaccinated patients with active rheumatoid arthritis, indicating the biological and clinical activity of this therapy.

Further phase I/II studies are under way in the fields of allergic diseases (allergic asthma), autoimmunity (Crohn’s disease, diabetes type 1, rheumatoid arthritis, and multiple sclerosis), and transplantation medicine (kidney transplantation) (https://clinicaltrials.gov).

A multitude of protocols has been developed to generate human tolerogenic DCs that can be tailored to induce specific tolerance. These innovative and attractive tools represent a promising therapeutic approach to treat inflammatory, autoimmune and allergic diseases, and transplant rejections. However, there is a high need to define optimal vaccination protocols and to identify the underlying immune mechanism of tolerance induction by human DCs in more detail. In this context, high-throughput approaches, e.g., in form of genomics and proteomics, will be of great help to analyze critical pathways contributing to programming and function of human tolerogenic DCs (122). Furthermore, next-generation tolerogenic DC vaccines should be integrated into future combinatorial immunotherapy regimes, including biologicals, nanoparticles, and *in vivo* targeting of DCs. So, it was demonstrated that combination of tolerogenic DCs with CTLA-4Ig strengthen their tolerogenic effect (123).

## Conclusion

Dendritic cells are the most potent professional antigen-presenting cells of the immune system and bridge innate and adaptive immunity by interacting with a large number of different cell types, thereby initiating and regulating adaptive immune responses. Hence, DCs are promising targets for immunotherapy either for initiating immunity as for example desired for the clearance of pathogens or antitumor immunotherapy or for the objective to alleviate unwanted and excessive immune responses in allergic and autoimmune disorders. Multiple *ex vivo* protocols have been established to induce stable tolerogenic human DCs exhibiting numerous different mechanisms to dampen immune responses. Those DCs may be used for antigen-specific induction of tolerance *in vivo*, which would be exceptionally beneficial for the therapy of allergic and autoimmune disease or in transplantation medicine. Progress in the fields of improved immunization protocols, genome editing, expression of recombinant proteins, and nano-dimensional drug delivery may contribute to overcome obstacles and to open up new unexpected approaches to improve the promising therapeutic option of DC vaccination for the future.

## Author Contributions

All authors wrote and critically read the manuscript.

## Conflict of Interest Statement

The authors declare that the research was conducted in the absence of any commercial or financial relationship that could be construed as a potential conflict of interest.
